# Silibinin alleviates inflammation and induces apoptosis in human rheumatoid arthritis fibroblast-like synoviocytes and has a therapeutic effect on arthritis in rats

**DOI:** 10.1038/s41598-018-21674-6

**Published:** 2018-02-19

**Authors:** W. W. Tong, C. Zhang, T. Hong, D. H. Liu, C. Wang, J. Li, X. K. He, W. D. Xu

**Affiliations:** 1 Department of Joint Bone Disease Surgery, Changhai Hospital, Second Military Medical University, 168 Changhai Road, Shanghai, 200433 China; 2 Department of Endocrinology, Drum Tower Hospital Affiliated to Nanjing University Medical School, No 321 Zhongshan Road, Nanjing, China; 3 Department of Orthopedics, First People’s Hospital, Aksu Prefecture, Xinjiang, China

## Abstract

Silibinin, a natural polyphenolic flavonoid, possesses anti-oxidant, anti-inflammation and anti-cancer properties. The present study was designed to investigate the effects of silibinin on rheumatoid arthritis (RA) pathogenesis-related cells and collagen-induced arthritis (CIA) and further explore the potential underlying mechanisms. Our results showed that silibinin suppressed cell viability and increased the percentage of apoptotic RA-fibroblast-like synoviocytes (FLS). Furthermore, the production of inflammatory cytokines in RA-FLS and a CIA rat model was effectively inhibited by silibinin. Silibinin also induced macrophage M2 polarization in RAW264.7 cells. We further demonstrated that silibinin inhibits Th17 cell differentiation *in vitro*. The nuclear factor kappa B (NF-κB) pathway was suppressed in RA-FLS. In addition, Sirtuin1 (SIRT1) was decreased after silibinin treatment, and RA-FLS transfection with a short hairpin RNA (shRNA) of SIRT1 enhanced silibinin-induced apoptosis. Autophagy was markedly decreased in a dose-dependent manner following silibinin treatment. These findings indicate that silibinin inhibited inflammation by inhibiting the NF-κB pathway, and SIRT1 may participate in silibinin-induced apoptosis. Silibinin also inhibited autophagy in RA-FLS. Thus, silibinin may be a potential therapeutic agent for the treatment of RA.

## Introduction

Rheumatoid arthritis (RA) is an autoimmune disease characterized by synovial inflammation and joint destruction^1^. The proliferation of fibroblast-like synoviocytes (FLS) as well as the infiltration of activated immune cells (including lymphocytes and macrophages) lead to persistent synovial inflammation and progressive joint destruction^2,3^.

Aggressive FLS produce large amounts of pro-inflammatory cytokines and proteolytic enzymes, ultimately leading to the destruction of joint cartilage and bone^4^. In addition, immune cells, such as T lymphocytes and macrophages, produce pro-inflammatory mediators, which induce chronic inflammation in RA. RA has been recognized as a T-helper (Th)-1 and Th-17 lymphocyte-mediated disease, and Th17, a T cell-effector lineage distinct from Th1 and Th2, has renewed interest in a pro-inflammatory role for T cells in RA^5–7^. Additionally, macrophages are crucial for inflammatory processes associated with synovial hyperplasia in RA^8^. Thus, inhibiting the synthesis of inflammatory cytokines and inducing the apoptosis of proliferative FLS are therapeutic targets for RA.

Silibinin, a natural polyphenolic flavonoid, is the major active constituent in silymarin extracted from milk thistle (*Silybum marianum*)^9^. Silibinin reportedly possesses strong anti-oxidant, anti-inflammation and anti-cancer properties^10–13^. We hypothesize that silibinin exerts strong efficacy in attenuating inflammation and inducing apoptosis in RA.

Sirtuins (SIRTs) are a family of NAD+-dependent histone deacetylases modulating gene expression. The most extensively studied member of the mammalian sirtuin family is SIRT1, which plays diverse roles in cell metabolism, survival, ageing, and inflammation^14,15^. Recent studies have demonstrated higher SIRT1 protein expression in whole synovial tissue and cultured FLS from the joints of patients with RA that that from patients with OA. Downregulation of SIRT1 was found to reduce the expression of pro-inflammatory mediators and induce the apoptosis of FLS^16^. Silencing of SIRT1 also reduce proliferation and leukocytic adhesion to RA-FLS^17^. The importance of SIRT1 for the induction of apoptosis has made this protein a novel target for RA therapy.

Autophagy removes defective cellular organelles and protein aggregates for organelle turnover and is associated with cell survival^18^. Increasing evidence suggests that enhanced autophagy correlates with reduced apoptosis in RA^19,20^. Thus, autophagy may be a potential therapeutic target in RA.

In this study, we evaluated the effects of silibinin on cell viability and inflammatory cytokine secretion in RA-FLS. We also investigated the effects of silibinin on macrophage M1/M2 polarization. The effects of silibinin on Th17 differentiation were further analysed. Collagen-induced arthritis (CIA) rats were used to examine the therapeutic effects of silibinin *in vivo*. Moreover, nuclear factor kappa B (NF-κB) signalling, SIRT1 signalling and autophagy were studied to explore the mechanisms underlying the therapeutic properties of silibinin.

## Results

### Silibinin decreases cell viability in RA-FLS

**Figure 1:**
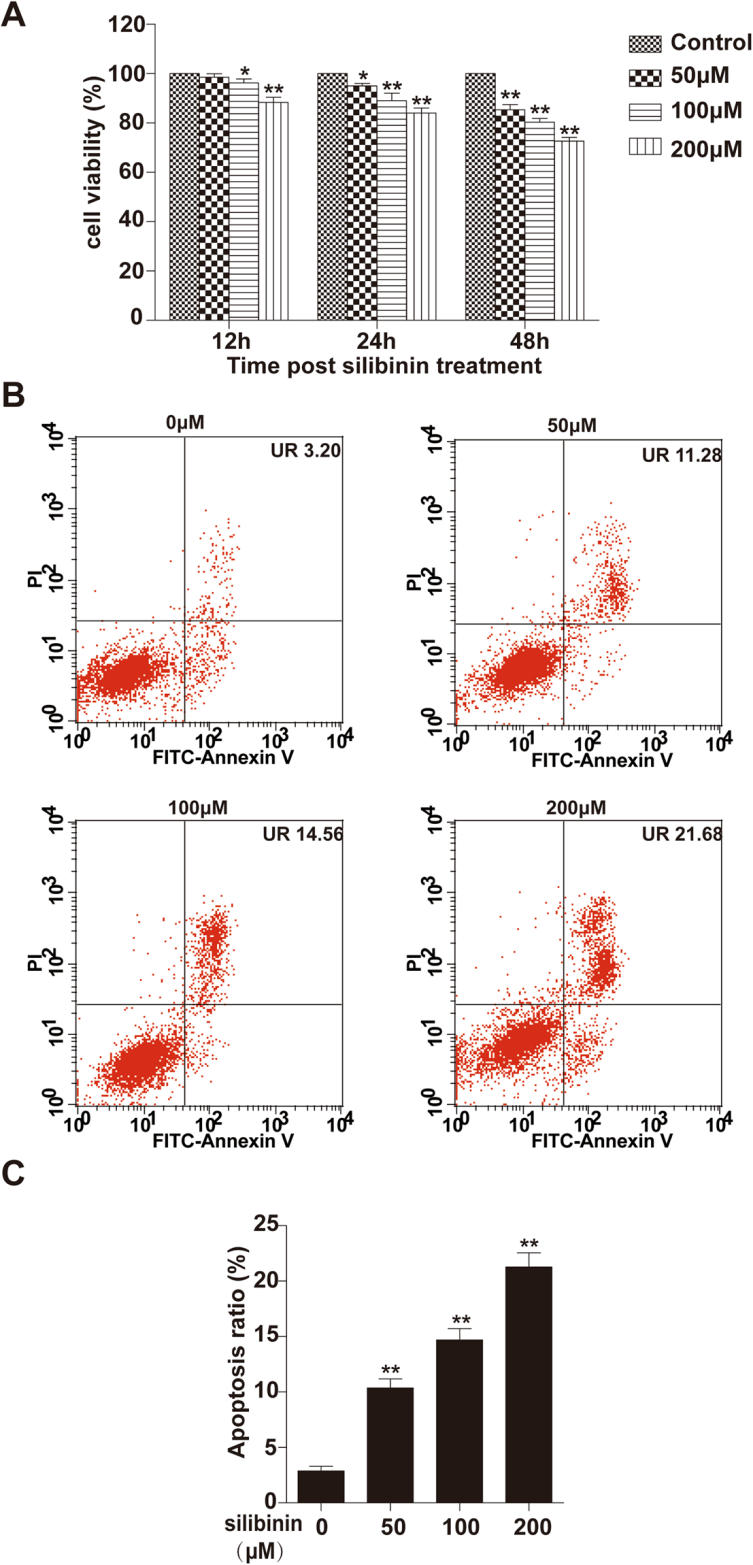
*In vitro* treatment of human RA-FLS inhibits cell viability and induces apoptosis in a dose- and time-dependent manner. (**A**) Cells were treated with various concentrations (0, 50, 100, and 200 μM) of silibinin for 12 h, 24 h and 48 h, and then cell viability was determined using the CCK-8 assay as described in the Materials and Methods section. The result is represented as control% compared to the control group. (**B**) Cells were treated with various concentrations of silibinin (0, 50, 100, and 200 μM) for 48 h and then harvested for apoptosis analysis by staining with Annexin V-FITC and PI. The upper right (UR) quadrant indicates the percentage of late apoptotic cells. (**C**) Quantitative analysis of the apoptosis rate. Data are presented as the mean ± SD from three independent experiments. *P < 0.05, **P < 0.01 compared to the 0 μM silibinin group.

We performed a Cell Count Kit-8 (CCK8) assay to determine the effects of silibinin on RA-FLS viability. The cells were treated with silibinin at various concentrations (0, 50, 100, and 200 μM) for different durations (12, 24, and 48 h). After treatment with silibinin, RA-FLS showed a dose- and time-dependent decrease in cell viability (Fig. 1A). Treatment with silibinin for 48 h led to a significant decrease in cell viability. Thus, treatment for 48 h was used in subsequent apoptosis experiments.

### Silibinin induces apoptosis in RA-FLS

To examine whether the reduction in cell viability with silibinin treatment was related to the induction of apoptosis, RA-FLS were treated with various concentrations (0, 50, 100, and 200 μM) of silibinin for 48 h. We performed flow cytometry analysis with Annexin V and propidium iodide to measure the proportion of apoptotic cells. Silibinin induced the apoptosis of RA-FLS in a concentration-dependent manner (Fig. 1A and B).

### Silibinin alleviates inflammation in RA-FLS by inhibiting NF-κB signalling

**Figure 2:**
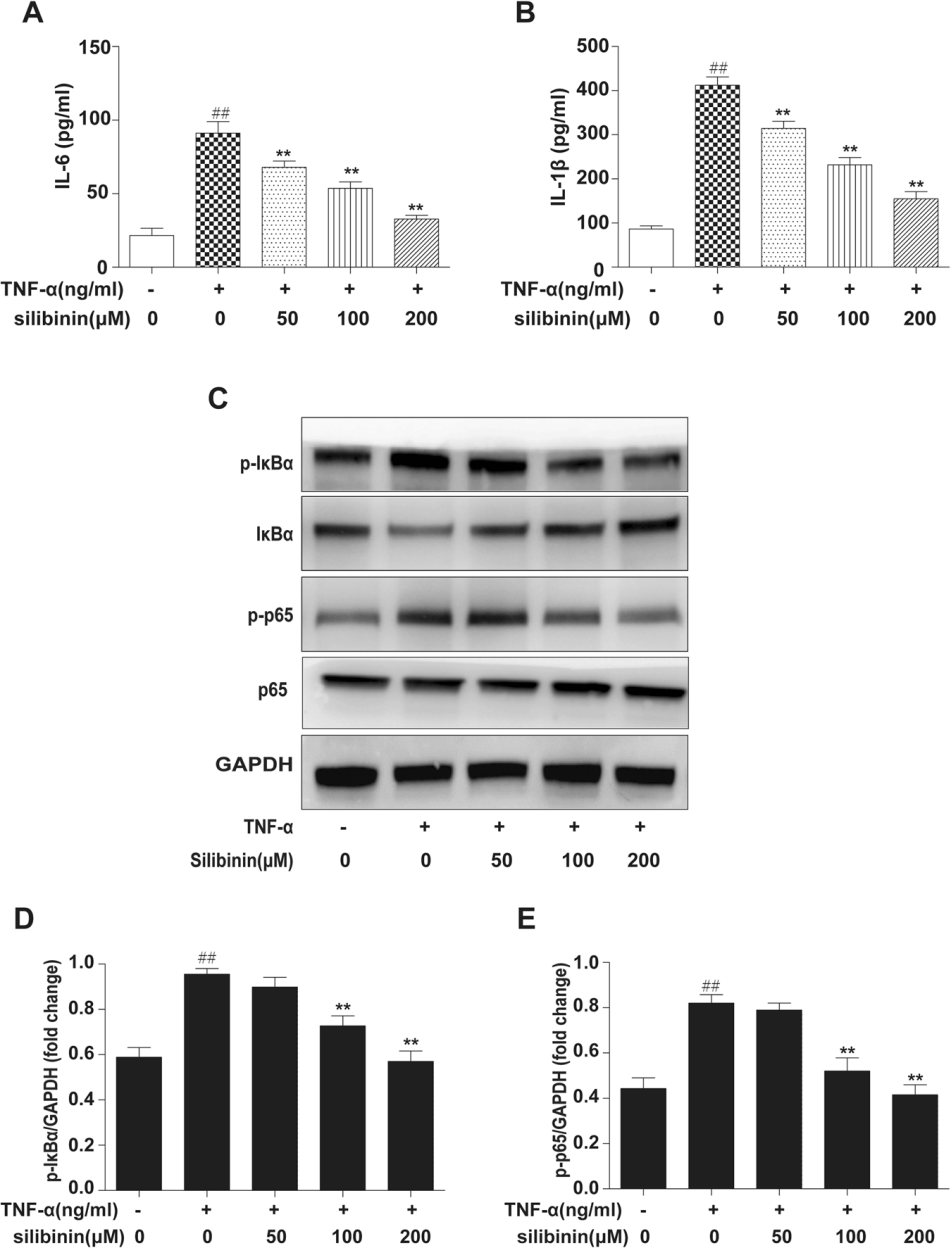
Effects of silibinin on TNF-α-induced cytokine production and activation of NF-κB in RA-FLS. RA-FLS were pre-treated with or without different concentrations (50, 100, and 200 μM) of silibinin and then exposed to TNF-α (10 ng/ml) for 12 h. (**A**) IL-6 and IL-1β levels in cultured cell supernatants were measured by ELISA. (**B**) Phospho-IκBα, IκBα, phospho-p65, p65 and GAPDH were determined by Western blot analysis. The intensity of phospho-IκBα and phospho-p65 was determined by densitometry using ImageJ software and normalized to the control (p-IκBα/GAPDH, p-p65/GAPDH). Data are representative of three independent experiments (mean ± SD). ^##^P < 0.01 compared to the group without TNF-α. *P < 0.05, **P < 0.01 vs treatment with TNF-α alone.

IL-6 and IL-1β are critical pro-inflammatory cytokines in RA pathogenesis. To investigate the anti-inflammatory functions of silibinin, RA-FLS were pre-treated with or without different concentrations of silibinin (0, 50, 100, and 200 μM) for 2 h and then exposed to TNF-α (10 ng/ml) for 12 h. RA-FLS stimulated with TNF-α (10 ng/ml) for 12 h resulted in increased IL-6 and IL-1β production. Silibinin markedly inhibited TNF-α-induced IL-6 (Fig. 2A) and IL-1β (Fig. 2B) production in a dose-dependent manner.

The secretion of inflammatory cytokines in the synovium is primarily regulated by NF-κB signalling, and these inflammatory cytokines provide positive feedback to NF-κB^21^. To gain insight into the mechanisms underlying the therapeutic effects, we used Western blot analysis to examine whether silibinin inhibits the NF-κB signalling pathway (Fig. 2C). The results indicated that TNF-α-induced phosphorylation of NF-κB p65 and IκBα was suppressed by silibinin in a dose-dependent manner (Fig. 2D and E).

### Silibinin induces Raw264.7 macrophage M2 polarization

**Figure 3:**
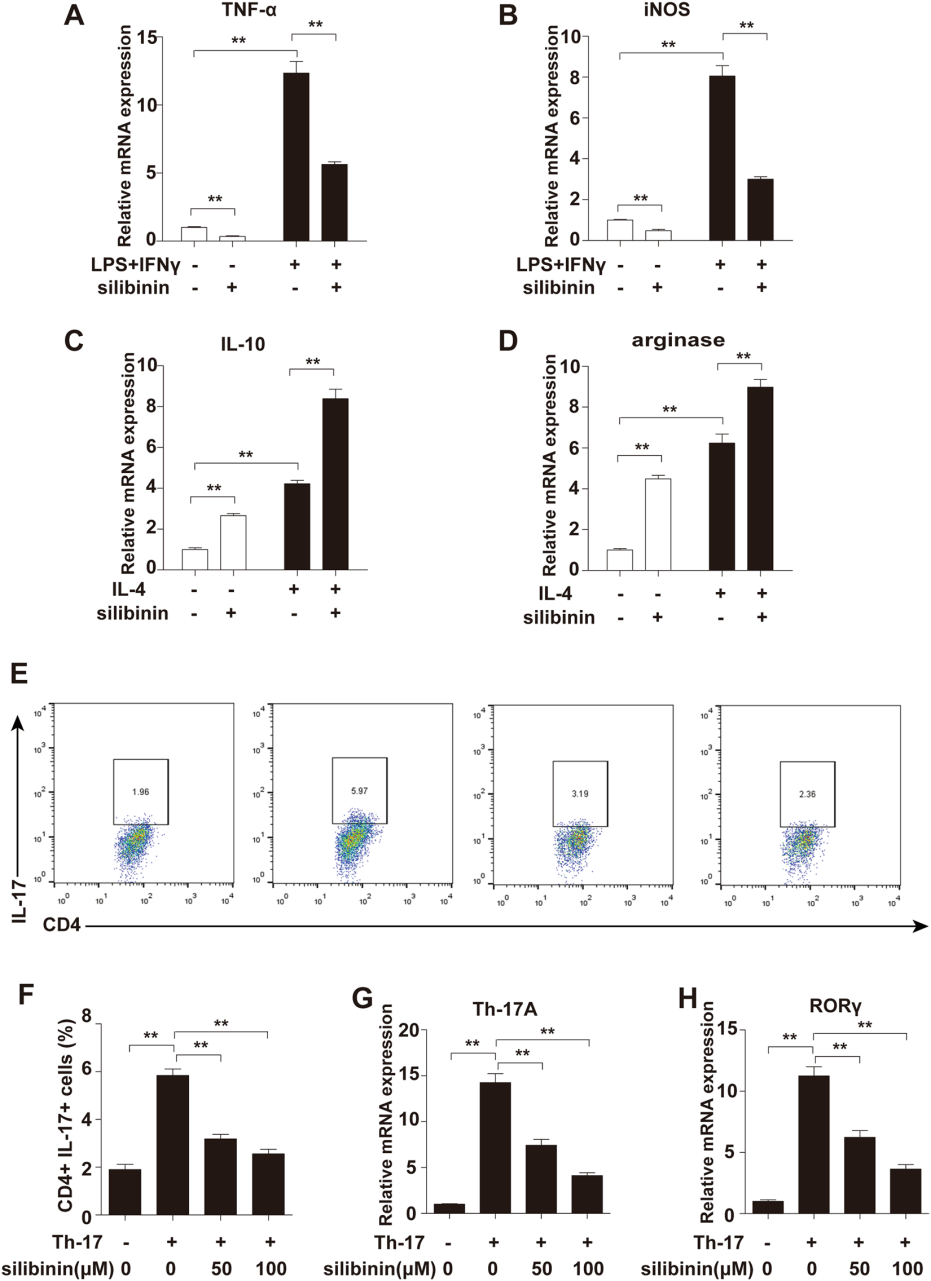
Silibinin induces Raw264.7 macrophages M2 polarization and inhibits Th17 cell differentiation *in vitro*. Raw264.7 macrophages were treated with silibinin (100 µM) for 24 h. To induced M1 polarization, the macrophages were incubated with LPS (1 µg/ml) and IFN γ (20 ng/ml) for 12 h. To induced M2 polarization, the macrophages were incubated with IL-4 (20 ng/ml, 12 h). The mRNA expression levels of (**A**) TNF-α, (**B**) iNOS, (**C**) IL-10 and (**D**) arginase were quantified by qPCR and normalized versus β-actin. Naive CD4^+^ T cells were purified from the spleens of DBA/1 mice and cultured under Th17-polarizing conditions for 3 days in the presence of silibinin (0, 50, and100 μM), and (**E**,**F**) the frequency of IL-17^+^CD4^+^ T cells was analyzed by flow cytometry. The flow cytometric profiles are representative of three independent experiments with similar results. The mRNA levels of (**G**) IL-17 and (**H**) RORγ were measured by real-time PCR. Data are presented as mean ± SD from three independent experiments. *P < 0.05, **P < 0.01.

Macrophages of the M1 phenotype act as pro-inflammatory mediators whereas those of the M2 phenotype suppress inflammation. To investigate the effects of silibinin on macrophage polarization, we detected changes in M1/M2 macrophage markers in RAW264.7 cells (a murine macrophage-like cell line) treated with silibinin. As shown in Fig. 3A and B, silibinin suppressed the expression of M1 macrophage markers, including TNF-α and inducible NO synthase (iNOS). Furthermore, silibinin potentiated the expression of M2 macrophage markers, including IL-10 and arginase (Fig. 3C and D).

M1 macrophage markers were significantly elevated in response to LPS and IFNγ. In addition, M2 macrophage markers were significantly elevated in response to IL-4. Silibinin suppressed the upregulation of TNF-α and iNOS mRNA expression induced by LPS and IFNγ (Fig. 3A and B). Furthermore, silibinin significantly enhanced the expression of M2 macrophage markers induced by IL-4 (Fig. 3C and D).

### Silibinin inhibits Th17 cell differentiation ***in vitro***

To evaluate whether silibinin exerts any direct effects on Th17 cell differentiation *in vitro*, naive CD4^+^ T cells were purified from the spleens of DBA/1 mice and cultured under Th17-polarizing conditions for 3 days with or without silibinin, and the frequency of IL-17^+^CD4^+^ T cells was analysed by flow cytometry. As shown in Fig. 3E and F, silibinin reduced the proportions of IL-17-expressing T cells in CD4^+^ cell populations in a dose-dependent manner. Furthermore, the expression of Th17 cell differentiation-associated genes, including IL-17 and RORγ (a transcription factor for Th17 differentiation), was significantly suppressed by silibinin (Fig. 3G and H). These results suggested that silibinin may alleviate RA by suppressing the differentiation of Th17 cells.

### Role of SIRT1 in silibinin-induced apoptosis in RA-FLS

**Figure 4:**
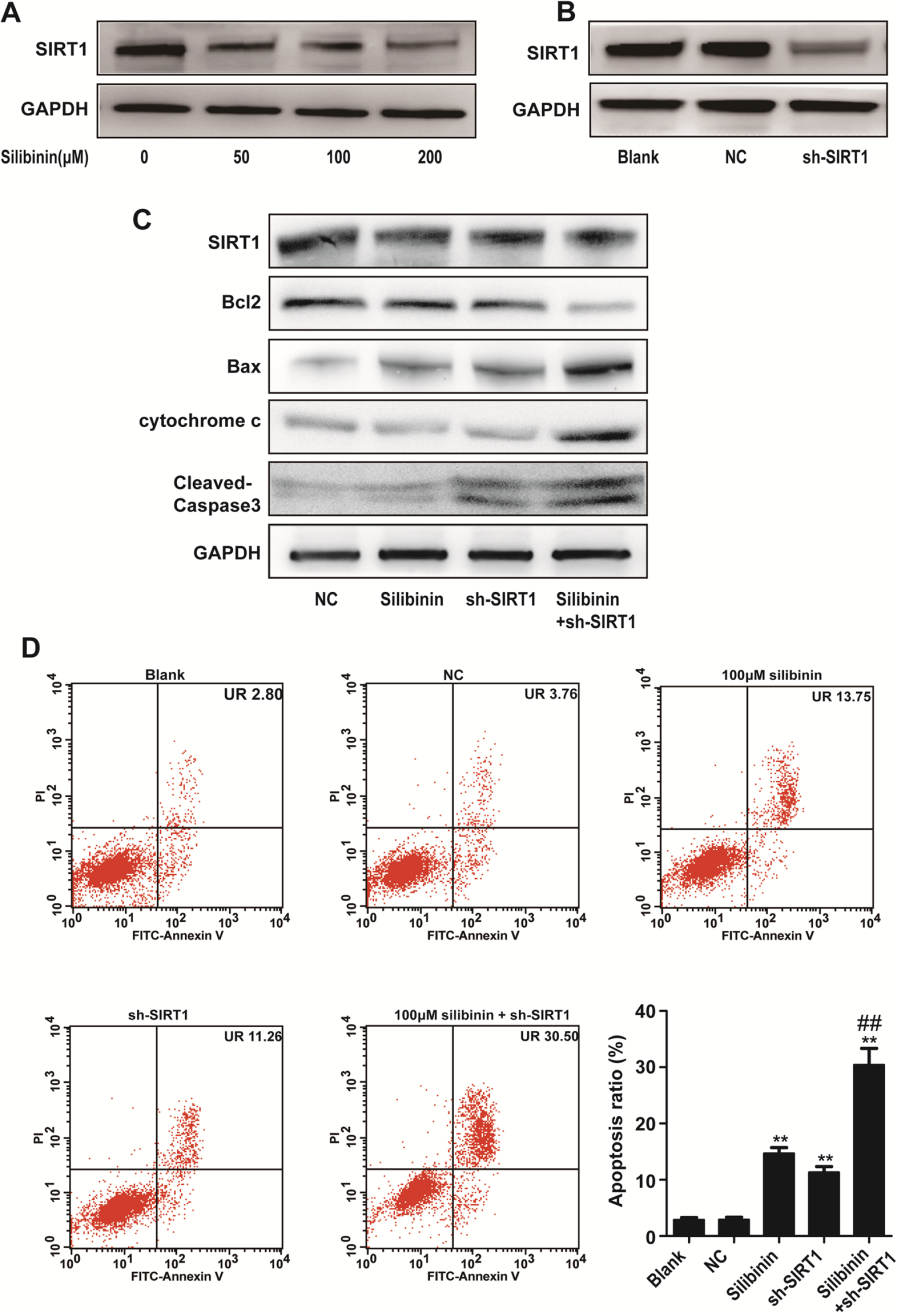
Role of SIRT1 in silibinin-induced apoptosis in RA fibroblast-like synoviocytes. (**A**) RA-FLS was treated with different concentrations (50, 100, and 200 μM) of silibinin for 48 h, and the expression of SIRT1 was analysed in the cell extract by Western blotting. (**B**) Effect of SIRT1-shRNA (sh-SIRT1) or control-shRNA (NC) transfection on SIRT1 protein levels in RA-FLS cells. SIRT1 expression levels were estimated by Western blotting. (**C**) Effect of sh-SIRT1 combined with silibinin (100 μM, 48 h) on the expression of SIRT1, Bcl2, Bax, Cytochrome c and Cleaved caspase-3. GAPDH was used as a loading control. The intensity was determined by densitometry using ImageJ software and normalized to the loading control. (**D**) Silibinin and sh-SIRT1 induced apoptosis in RA-FLS. The apoptosis levels were examined by flow cytometry. Data are presented as the mean ± SD from three independent experiments. *P < 0.05, **P < 0.01 compared to the control group. ^##^P < 0.01 compared to the silibinin-treated group.

The expression of SIRT1 in RA-FLS was decreased after silibinin treatment. Given that SIRT1 plays an important role in regulating apoptosis. We next evaluated the role of SIRT1 in silibinin-induced apoptosis in RA-FLS. RA-FLS were exposed to silibinin at various concentrations (50, 100, and 200 μM) for 48 h, and SIRT1 protein expression was estimated by Western blot. Indeed, SIRT1 expression was reduced in a concentration-dependent manner (Fig. 4A).

We next established a SIRT1 short hairpin RNA (SIRT1-shRNA)-mediated silencing system. Transfection with shRNA decreased the expression of SIRT1 (Fig. 4B), and the effect was significantly enhanced after co-treatment with silibinin (Fig. 4C). As shown in Fig. 4C, the mitochondrial apoptotic pathway-related proteins Bax and cytosolic cytochrome c were upregulated after silibinin or SIRT1-shRNA treatment alone and were further upregulated by silibinin and SIRT1-shRNA co-treatment. In contrast, Bcl2 was downregulated during the process. Furthermore, caspase activity (determined by Western blot for cleaved caspase-3) was substantially enhanced after silibinin or SIRT1-shRNA treatment alone and further upregulated by silibinin and SIRT1-shRNA co-treatment. The band intensities were normalized to GAPDH (Supplementary Fig. 1). Knockdown of SIRT1 induced apoptosis in the absence of silibinin. Furthermore, the specific reduction of SIRT1 expression upon transfection with shRNA-enhanced silibinin-induced apoptosis in RA-FLS (Fig. 4D). Together, these data indicated that the inhibition of SIRT1 signalling sensitizes silibinin-induced apoptosis.

### Silibinin inhibits autophagy in RA-FLS

**Figure 5:**
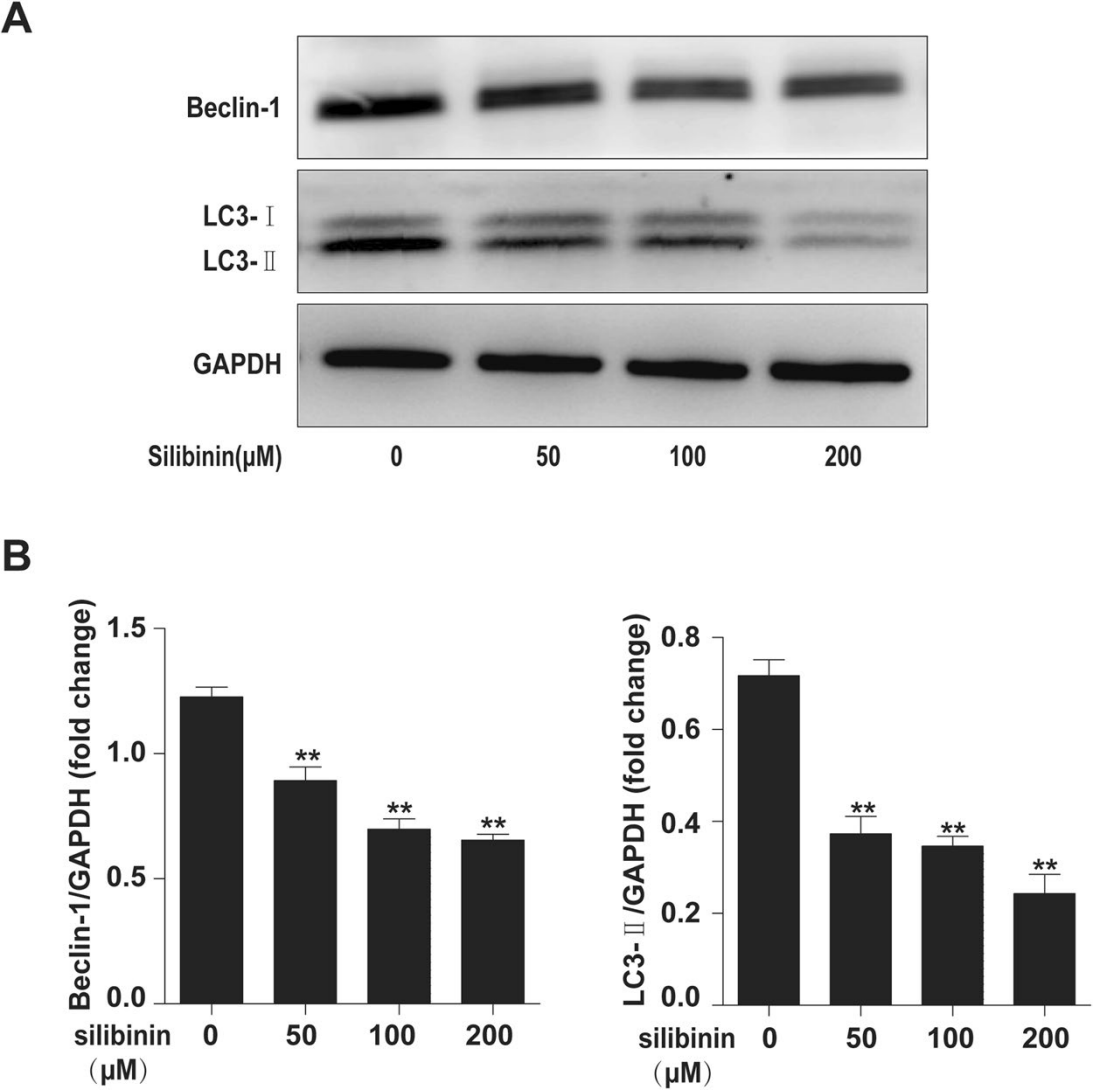
Silibinin inhibits autophagy in RA-FLS. (**A**) RA-FLS were treated with different concentrations (50, 100, and 200 μM) of silibinin for 48 h, and Beclin-1 and LC3 expression was analysed in the cell extracts by Western blotting. (**B**) The intensity of Beclin-1 and LC3 was determined by densitometry using ImageJ software and normalized with the loading control (SIRT1/GAPDH). Data are presented as the mean ± SD from three independent experiments. *P < 0.05, **P < 0.01 compared to the 0 μM silibinin group.

To determine the effects of silibinin on autophagy, RA-FLS were treated with various concentrations (50, 100, and 200 μM) of silibinin for 48 h. LC3II and beclin-1 protein levels decreased markedly in a dose-dependent manner following silibinin treatment (Fig. 5A and B).

### Silibinin attenuates the degree of arthritis caused by type II collagen in rats

**Figure 6:**
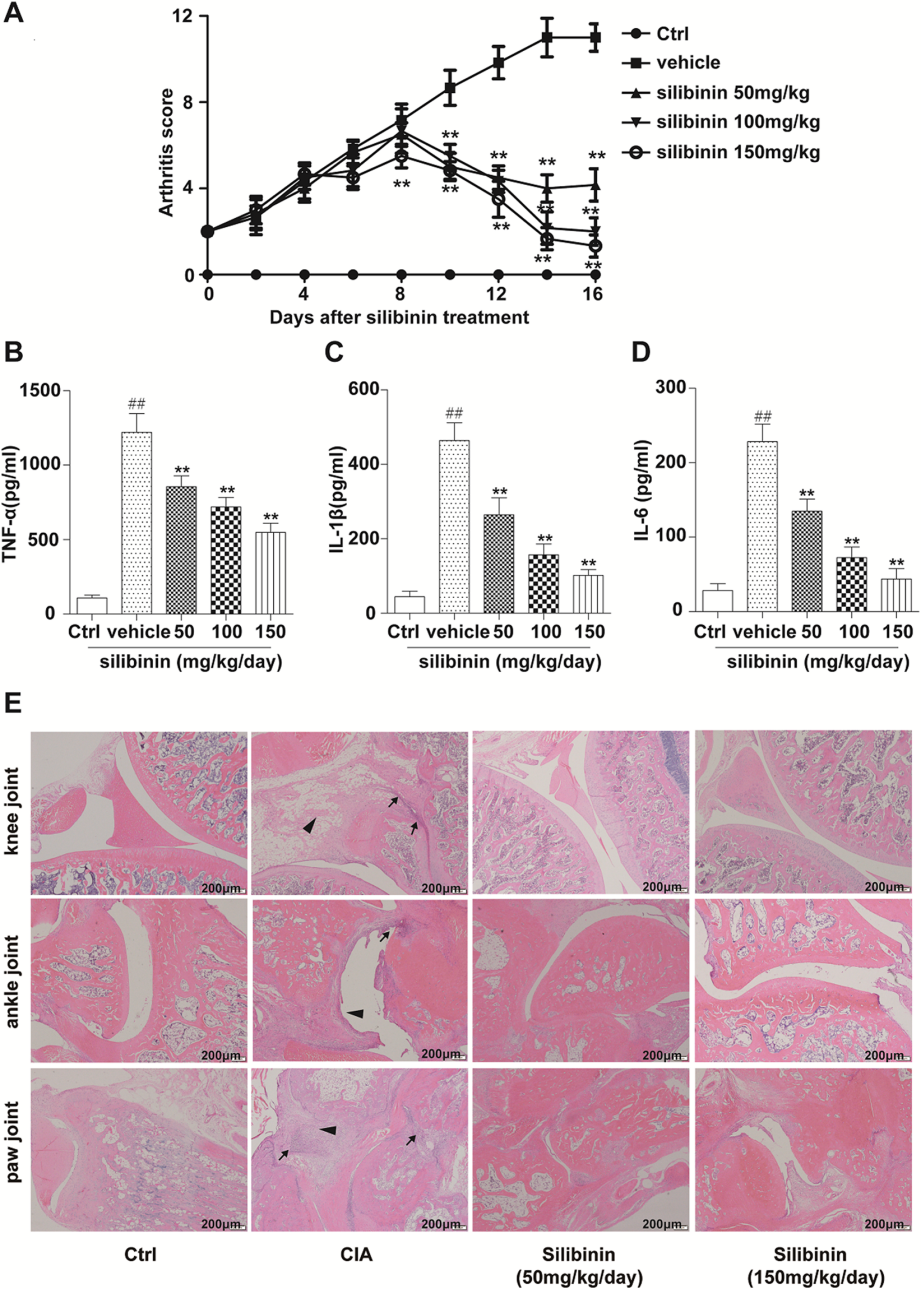
Effects of silibinin on CIA. Rats were randomly divided into 5 groups, with six rats in each group: without CIA (Ctrl), CIA+ vehicle, CIA+ silibinin 50 mg/kg/day, CIA+ silibinin 100 mg/kg/day, and CIA+ silibinin 150 mg/kg/day. (**A**) Treatment with silibinin led to dose-dependent improvements in the clinical arthritis score compared with vehicle. Silibinin also reduced cytokine levels in the sera of CIA rats. (**B**) TNF-α, (**C**) IL-1β and (**D**) IL-6. (**E**) Pannus formation (arrowheads) and infiltration of inflammatory cells (thin arrows) were attenuated by two weeks treatment of silibinin. Data are expressed as the mean ± SD. ^##^P < 0.01 compared to the Ctrl group, *P < 0.05, **P < 0.01 represent significant differences when compared with vehicle treatment.

Having shown potential anti-inflammation effects *in vitro*, we next examined the therapeutic effects of silibinin *in vivo* using a type II collagen-induced arthritis. Silibinin treatment resulted in a significant reduction in joint inflammation, as evidenced by a significant decrease in arthritis scores compared with those of rats without silibinin treatment (Fig. 6A). To investigate whether silibinin modulates the inflammatory process through the regulation of cytokine secretion *in vivo*, we measured the serum levels of TNF-α, IL-6, and IL-1β in rats by ELISA. Compared with the vehicle-treated group, TNF α, IL-1β and IL-6 levels in the silibinin-treated group were significantly decreased (Fig. 6B–D). Histologically, the knee, ankle and paw joints of vehicle-treated CIA rats showed severe pannus formation, inflammatory cell infiltration and bone erosion. The histologic changes were attenuated by silibinin treatment. Representative images of the changes in the joints are shown in Fig. 6E.

## Discussion

Silibinin has been widely used for the treatment of hepatic disorders and has also been shown favourable results for cancer prevention and treatment^22–24^. Due to its anti-inflammation and anti-proliferation features, in the present study we explored the effects and underlying mechanisms of silibinin in RA. The results showed that silibinin inhibited inflammation and induced apoptosis in RA-FLS and demonstrated therapeutic effects *in vivo* in a CIA rat model. Silibinin also induced macrophage M2 polarization in RAW264.7 cells. We further demonstrated that silibinin inhibits Th17 cell differentiation *in vitro*. Our exploration of the underlying mechanisms showed that NF-κB signalling pathways were involved in the anti-inflammation effects of silibinin. SIRT1 may participate in silibinin-induced apoptosis. Inhibition of autophagy may contribute to the anti-inflammation and apoptosis-induced effects of silibinin.

RA is a chronic inflammatory disorder characterized by the release of cytokines such as TNF-α, IL-1β, IL-6 and IL-8^3^. RA-FLS, T lymphocytes and macrophages play key roles in RA by producing cytokines that perpetuate inflammation^1,4,5,8^. Thus, the primary aim of RA treatment is to control inflammatory cytokine secretion in pathogenesis-related cells. Findings from a number of studies have indicated that silymarin (whose main active constituent is silibinin) has the ability to inhibit T cell proliferation and pro-inflammatory cytokine secretion *in vitro*^25–28^. Additionally, previous studies have demonstrated that silibinin inhibits macrophage activation by blocking p38 MAPK or NF-κB signalling pathways in isolated mouse peritoneal macrophages or RAW 264.7 cells^29,30^. In this study, we found that silibinin significantly inhibited TNF-α-induced production of IL-6 and IL-1β in RA-FLS. Moreover, silibinin inhibited TNF-α-induced NF-κB pathway activation in RA-FLS by attenuating IκBα and NF-κB p65 phosphorylation. Silibinin also induced the M2 polarization of RAW264.7 macrophage cells. We further showed that silibinin inhibits Th17 cell differentiation *in vitro*. Next, an animal experiment demonstrated that silibinin significantly alleviated the swelling of paws and decreased cytokine secretion. All of our data indicated that silibinin suppresses inflammation reactions in RA. Previous studies have shown that Tregs mediate immune tolerance in type II collagen-induced RA^31,32^. The effects of silibinin on Treg cells are controversial^33,34^ and require further investigation. Granulocyte macrophage colony stimulating factor (GM-CSF) has been reported to promote Treg expansion and play a major role in suppressing autoimmune diseases^35–39^. Moreover, GM-CSF expression is increased after silibinin treatment^40^. Therefore, we anticipate that GM-CSF may synergize with silibinin to ameliorate CIA, although this hypothesis requires further investigation.

Apoptosis is generally recognized as a form of programmed cell death through which damaged and aged cells are eliminated^41^. RA-FLS exhibit resistance to apoptosis compared with normal fibroblasts as well as the invasion of synovial tissues, leading to progressive destruction of the joints^42^. Our results demonstrated that silibinin suppressed RA-FLS viability and induced apoptosis in a dose-dependent manner. Findings from a number of studies have indicated that SIRT1 is an anti-apoptotic protein in RA-FLS. SIRT1 overexpression promotes apoptotic resistance, and silencing of SIRT1 induces RA-FLS apoptosis^16,43^. In this study, a SIRT1 knockdown experiment showed that inhibition of SIRT1 sensitized silibinin-induced apoptosis. Moreover, silibinin downregulated the expression of SIRT1 and exerted effects on several apoptosis-related proteins. Thus, our studies suggest that silibinin may induce RA-FLS apoptosis via SIRT1 downregulation. However, resveratrol reportedly induces the apoptosis of MH7A cells by upregulating SIRT1 expression^44^. Effects on SIRT1 expression may vary according to cell type, or SIRT1 expression may affected by many other factors. Moreover, the role of SIRT1 in arthritis models is controversial. Deletion of SIRT1 was found to suppress collagen-induced arthritis but aggravate serum transfer arthritis^45,46^. Thus, the exact function of SIRT1 in the apoptosis of RA-FLS requires further exploration.

Autophagy is a catabolic process through which damaged or obsolete organelles and proteins are delivered to lysosomes for proteolysis to maintain cell homeostasis^47^. Autophagy has been shown to engage in a complex interplay with apoptosis, and the inhibition of autophagy reportedly induces apoptosis in several cancer cell types^48,49^. Moreover, autophagy inhibitors effectively inhibit the immune activation function of RA-FLS medicated by IL-6, and anti-apoptosis effects induced by IL-17 are restored by autophagy inhibition^50,51^. Our results showed that silibinin suppressed autophagy in a dose-dependent manner. Thus, silibinin may exert its anti-inflammation and apoptosis-induced effects by inhibiting autophagy.

In conclusion, silibinin inhibited the production of inflammatory cytokines and induced apoptosis in RA pathogenesis-related cells. An animal model further confirmed the therapeutic function of silibinin. The anti-inflammation effects of silibinin might be attributable to its inhibition of NF-κB signalling. Furthermore, SIRT1 may play an important role in silibinin-induced apoptosis. In addition, our results revealed that silibinin suppressed autophagy in RA-FLS. These findings suggest that silibinin is a potential candidate for RA therapy.

## Materials and Methods

### Isolation and culture of cells

Synovial tissues were obtained from active RA patients who were undergoing joint replacement according to the revised criteria of the American College of Rheumatology. FLS were isolated from synovial tissues by enzymatic digestion as previously described^52^. The study was approved by the Medical Ethics Committee of Changhai Hospital, and informed consent was obtained from all patients. We confirmed that all methods were performed in accordance with relevant guidelines and regulations. RA-FLS were grown in DMEM/F12 containing 10% foetal bovine serum (FBS) and supplemented with 100 µg/ml streptomycin and 100 U/ml penicillin in a humidified incubator at 37 °C under 5% CO_2_. Cells were used between passages 4 and 6. The Raw264.7 macrophage cell line was obtained from the Cell Bank of the Chinese Academy of Sciences (Shanghai, China) and grown in RPMI-1640 supplemented with FBS, streptomycin and penicillin. All reagents were from Gibco (Grand Island, NY).

### Cell Viability Assay

The Cell Count Kit-8 (YEASEN, Shanghai, China) was used to evaluate the effects of silibinin on cell viability. Cells (7 × 10^3^ cells/well) were seeded in 96-well plates. After culturing for 24 h, cells were rinsed with phosphate-buffered saline (PBS) and were treated with various concentrations (0, 50, 100, 200 μM) of silibinin (Sigma-Aldrich, St. Louis, MO, USA) for 12 h, 24 h and 48 h. 10 μl CCK8 solution was added to each well and incubated at 37 °C for 2 h. The absorbance of supernatant was measured at a 450-nm wave length using a microplate reader.

### Annexin V-FITC/PI staining experiment

Apoptosis detection with flow cytometry was carried out using the Annexin V-FITC/PI apoptosis detection kit (BD Biosciences, San Diego, CA, USA) according to manufacturer’s instructions. 3 × 10^5^ cells/well were seeded in the 6-well plate. After 48 h of indicated treatment, cells were washed twice with cold PBS and resuspended in the 1× binding buffer. Cells were then stained with Annexin V-FITC and PI solution for 15 min at room temperature in the dark, and analyzed by flow cytometry.

### Measurements of cytokines level in serum and culture supernatants by ELISA

Cytokines level were measured by enzyme-linked immunosorbent assay (ELISA) kits from R&D system (Minneapolis, MN, USA) according to the manufacturer’s instructions.

### T-helper cell differentiation ***in vitro***

Naive CD4^+^CD62L^+^ T cells (purity of cells >95%) were purified from the spleens of DBA/1 mice using a CD4^+^CD62L^+^ T Cell Isolation Kit II (Miltenyi Biotec, Bergisch Gladbach, Germany) and cultured in RPMI 1640 medium supplemented with 10% FBS, 100 IU/ml penicillin and 100 mg/ml streptomycin. To induce Th17 polarization, the purified cells were stimulated with plate-bound anti-CD3 antibodies (eBioscience, San Diego, CA, USA, 2 μg/ml), anti-CD28 antibodies (eBioscience, 2 μg/ml), TGF-β (2 ng/ml), IL-6 (20 ng/ml), anti-IFN-γ antibodies (10 μg/ml) and anti-IL-4 antibodies (10 μg/ml), with or without silibinin (0, 50, and 100 μM), for 72 h. All reagents were from PeproTech (Rocky Hill, NJ, USA). Activated cells were restimulated with PMA (20 ng/ml, Sigma-Aldrich) and ionomycin (500 ng/ml, Sigma-Aldrich) in the presence of brefeldin A (10 μg/ml) for 5 h before intracellular staining was performed. Cells were stained with phycoerythrin-conjugated anti-mouse IL-17 antibodies (BD Biosciences) and measured by flow cytometry.

### RNA extraction and real-time PCR

Total RNA was extracted by TRIzol reagent (Invitrogen) and cDNA was prepared using PrimeScript reverse transcription kit (TaKaRa, Tokyo, Japan) according to the manufacturers’ protocols. Real-time PCR amplification reactions were prepared with the SYBR Green PCR Kit (TaKaRa). The relative expression of each target gene was quantified by calculating Ct (threshold cycle) values and normalized by β-actin levels. Each sample was analyzed in triplicate. We used the following primer sets: for RAR-related orphan receptor gamma (Rorc), 5′-GACCCACACCTCACAAATTGA-3′(forward) and 5′-AGTAGGCCACATTACACTGCT-3′ (reverse); for interleukin 17 A (IL-17A), 5′-TTTAACTCCCTTGGCGCAAAA-3′ (forward) and 5′-CTTTCCCTCCGCATTGACAC-3′ (reverse).

### Western blot analysis

After silibinin treatment, cells were harvested in RIPA lysis buffer containing protease inhibitors (thermo scientifical) according to the manufacturer’s instructions. The protein concentration was determined using the BCA assay (Beyotime). Equal amounts of protein (20 ug) were separated by SDS-PAGE gels and electrophoretically transferred to PVDF membrane (Bio-Rad, USA). After blocking with 5% BSA for 2 h at room temperature, the membranes were probed with primary antibodies for 12–16 h at 4 °C. Then membranes were incubated with appropriate HRP (horseradish peroxidase)–labeled secondary antibodies for 2 h at room temperature. Immunoreactive protein bands were visualized by enhanced chemiluminescence (ECL, Amersham Pharmacia Biotech, USA), and GAPDH was used as a protein loading control. The density of the specific bands was quantified using imageJ software. Antibodies for SIRT1, NF-κB were purchased from Abcam (Cambridge, MA, USA). Antibodies for Bcl2, Bax, cytochrome c, Cleaved-Caspase3, GAPDH, HRP-conjugated secondary antibodies were purchased from Cell Signaling Technology (Beverly, MA, USA). Antibodies for Beclin and LC3 were from Novus (Littleton, CO, USA).

### shRNA knockdown studies

To generate an shRNA targeting SIRT1 (GenBank accession number NM_001142498.1), the sense strand (5′-GACGCTGTGGCAGATTGTTAT-3′) was cloned into a lentivirus expression vector. The reconstructed vector was then transfected into 293 T cells using Lipofectamine® reagent (Invitrogen; Thermo Fisher Scientific, Inc., USA). The lentiviral particles were collected at 48 h post-transfection. RA-FLS cells were cultured in 6-well plates at an inoculation density of 5 × 10^4^ cells/well and infected with a lentivirus containing SIRT1-shRNA or control-shRNA (NC). After incubation for 72 h, the infection efficiency was observed using fluorescence microscopy and analysed by qPCR and Western blotting.

### Induction of collagen-induced arthritis (CIA) and treatment

All animal experimental protocols were approved by the animal care and use committee of Changhai hospital and we confirmed that all methods were performed in accordance with the relevant guidelines and regulations. Female wistar rats (6–8 weeks) were purchased from SLRC Laboratory Animals (Shanghai, China). Bovine type II collagen (CII, Chondrex) was dissolved in 0.01 M acetic acid at a concentration of 2 mg/ml and frozen at −80 °C until use. Then, bovine CII was emulsified with an equal volume of complete Freund’s adjuvant (CFA, sigma). Wistar rats were immunized with the emulsion (containing 200 ug of CII) intradermally at the base of the tail. On day 21, a booster immunization was administered. The onset of arthritis occurs within 1 week after the second immunization. The arthritis score was determined by grading each paw from 0 to 4 based on erythema, swelling, and flexion of the joint and the sum of the scores for each mouse was calculated by adding the scores from the 4 individual paws^53^. CIA received silibinin (50, 100, 150 mg/kg/day, p.o.) or vehicle for 2 weeks since the onset of arthritis (arthritis scores >2). The arthritis score were measured three times per week.

### Statistical analysis

All values were expressed as the mean ± SD and analyzed by 1-way analysis of variance (ANOVA) followed by Bonferroni post hoc test for multiple comparisons after test of homogeneity of variances using authorized SPSS 19.0. A P-value of ≤0.05 was regarded as statistically significant.

## Electronic supplementary material

Supplementary Figure. 1
