# Supplementary material for: Silibinin alleviates inflammation and induces apoptosis in human rheumatoid arthritis fibroblast-like synoviocytes and has a therapeutic effect on arthritis in rats
Source: Sci Rep. 2018 Feb 19;8:3241. doi: 10.1038/s41598-018-21674-6 (PMC5818498; doi:10.1038/s41598-018-21674-6)

Silibinin alleviates inflammation and induces apoptosis in human rheumatoid arthritis fibroblast-like synoviocytes and has a therapeutic effect on arthritis in rats

Tong WW, Zhang C, Hong T, Liu DH, Wang C, Li J, He XK, Xu WD

**Supplementary Figure 1. The band intensities were normalized to GAPDH.** (A) The expression of SIRT1 in RA-FLS after treated with different concentrations (50, 100, and 200 μM) of silibinin for 48 h. (B) Effect of SIRT1-shRNA (sh-SIRT1) or control-shRNA (NC) transfection on SIRT1 protein levels in RA-FLS cells. (C-G) Effect of sh-SIRT1 combined with silibinin (100 μM, 48 h) on the expression of SIRT1, Bcl2, Bax, Cytochrome c and Cleaved caspase-3. GAPDH was used as a loading control. The intensity was determined by densitometry using ImageJ software and normalized to the loading control. Data are presented as the mean±SD from three independent experiments. *P<0.05, **P<0.01 compared to the control group. ##P<0.01 compared to the silibinin-treated group.


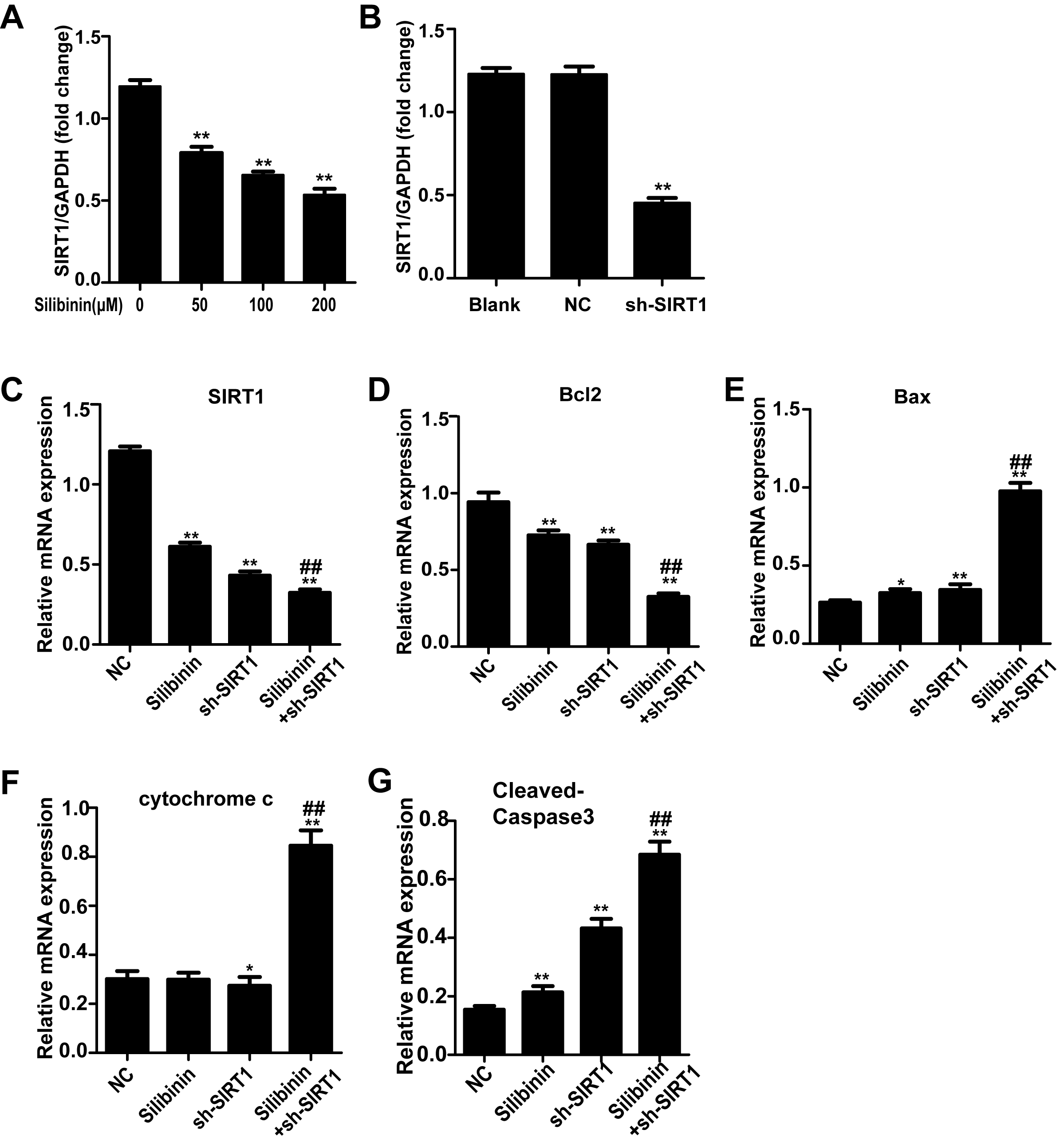

Supplement: Supplementary file 1 — Supplementary Figure. 1 [file 41598_2018_21674_MOESM1_ESM.docx]
